# GRANDPARENT–GRANDCHILD ASIAN AMERICAN REMINISCENCE: INTERGENERATIONAL AND CULTURAL REFLECTION

**DOI:** 10.1093/geroni/igae098.0628

**Published:** 2024-12-31

**Authors:** Ling Xu

**Affiliations:** The University of Texas at Arlington, Arlington, Texas, United States

## Abstract

Intergenerational solidarity between grandparents and grandchildren has been highlighted in Asian Families and it is beneficial for the health of grandparents. An intergenerational reminiscence program was developed to improve the well-being of Asian American older adults as well as generational bonding. This paper qualitatively reported the reflection of participant dyads based on the interviews at the end of program (n = 11). Older grandparents received 6 sessions of life-review with their grandchildren remotely or in person for approximately 1 hour each week for 6 weeks. At the end of the program, individual interviews were conducted with dyad participants. Braun and Clarke’s six-steps of thematic analysis were followed to examine the interview data. Thematic analysis of interview with grandparents revealed four themes: Family life (early childhood, intergenerational connection, family reunition through immigration); career and work (hard work, sacrifice personal life); major life turning points (immigration, baptism); stress and coping (grief/loss, resilience). Thematic analysis of qualitative data from grandchildren also yielded four themes: Unknown/unexpected family history; disagreement (life-work balance, being frugal); cultural respect, lasting legacy (always remembering the good part of life, take one thing at a time). Recommendations offered by the dyad participants included combining some topics to avoid overlapping, adding topics of spousal relationship or parenting, etc. The reflection from grandparent-grandchild dyad participants showed that intergenerational reminiscence program is promising in bonding the grandparent-grandchild connection with family history and cultural revisitation. Lessons learned and recommendations for future research on interventions for Asian American families are offered.

